# Breathing bad: increased risk for obstructive sleep apnea in current and former smokers

**DOI:** 10.1038/s41598-026-48908-2

**Published:** 2026-04-24

**Authors:** Markus Krüger, Christiane Pink, Hanyi Jiang, Antoine Weihs, Chia-Jung Busch, Christian Scharf, Thomas Bremert, Ralf Ewert, Anne Obst, Thomas Penzel, Ingo Fietze, Bernd Kordaß, Thomas Kocher, Reiner Biffar, Henry Völzke, Amro Daboul

**Affiliations:** 1 Poliklinik für zahnärztliche Prothetik, Alterszahnmedizin und Medizinische Werkstoffkunde, Universitätsmedizin Greifswald, Fleischmann-Str. 42, 17475 Greifswald, Germany; 2 Poliklinik für Zahnerhaltung, Parodontologie und Endodontologie, Universitätsmedizin Greifswald, Greifswald, Germany; 3 Klinik und Poliklinik für Psychiatrie und Psychotherapie, Universitätsmedizin Greifswald, Greifswald, Germany; 4 Klinik und Poliklinik für Hals-, Nasen-, Ohrenkrankheiten, Kopf- und Halschirurgie, Universitätsmedizin Greifswald, Greifswald, Germany; 5 Klinik und Poliklinik für Innere Medizin B, Universitätsmedizin Greifswald, Greifswald, Germany; 6 Interdisziplinäres Schlafmedizinisches Zentrum, Charité – Universitätsmedizin Berlin, Berlin, Germany; 7 Institut für Community Medicine - Department SHIP/ Klinisch-Epidemiologische Forschung, Universitätsmedizin Greifswald, Greifswald, Germany

**Keywords:** OSA, OSAS, Population-based, Smoking, Smoking status, Study of health in Pomerania, Diseases, Health care, Medical research, Risk factors

## Abstract

Obstructive sleep apnea (OSA) and smoking are both prevalent and impactful health risks. While smoking may contribute to OSA through inflammatory and neuromuscular pathways, population-based evidence on this relationship remains limited and inconsistent. A sample (*N* = 1,206) from the population-based Study of Health in Pomerania with complete overnight polysomnography and smoking assessment was investigated in a cross-sectional study for an association between OSA and former as well as recent smoking status. Regression models adjusted for Age and BMI were applied. Current smoking was significantly associated with increased apnea-hypopnea-index (AHI) severity (OR = 1.75, 95% CI [1.27; 2.41], *p* < .001), with stratified analyses confirming the effect across younger and older participants. Former smokers also showed significantly elevated AHI severity compared to never-smokers (OR = 1.76, 95% CI [1.27; 2.43], *p* < .001). Both current and former smoking were significantly associated with greater OSA severity in this population-based sample, even after accounting for age and BMI. The findings underscore the long-term respiratory consequences of smoking and highlight the need for integrated approaches in smoking cessation and OSA screening.

## Introduction

Obstructive sleep apnea (OSA) is characterized by intermittent episodes of partial or complete upper airway blockage during sleep, resulting in a cessation of airflow and a marked decrease in oxygen saturation. Much like smoking, sleep apnea is widely acknowledged to exert a considerable detrimental effect on health, both in the short and the long term^1,2^. Similar to smoking, OSA increases the risk for cardiovascular and metabolic diseases, stroke, and overall mortality substantially^2^. Like smoking, it is endemic – worldwide, an estimated one billion people are at risk, with 425 million most likely affected, resulting in a substantial economic burden on healthcare systems and society at large (e.g., the economic damage in Italy alone is estimated to range from €13.8 to €41.3 billion per annum)^3,4^. In the adult population in Pomerania, Germany, observed in this study, 46% of the tested participants were at risk and 6% fulfilled the diagnostic criteria for obstructive sleep apnea syndrome (OSAS) based on an increased apnea-hypopnea-index (AHI, the number of obstructions per hour during sleep) and self-reported daytime sleepiness^5^. Smoking may contribute to the onset and progression of OSA by altering sleep architecture, impairing upper airway neuromuscular reflexes, and causing upper airway inflammation^6^. A bidirectional effect is also possible: OSA may contribute to depression and reduced quality of life, which may, in turn, increase the likelihood of smoking initiation or hinder cessation efforts^6,7^.

In a recent meta-analysis by Zeng et al. a stable association between current smoking and OSA relying on overnight polysomnography (PSG) was found, which did not appear to be alleviated by smoking cessation^8^. However, this finding is not uniformly supported. For example, in a large patient study (*N* = 3,718) in Sao Paulo, Brazil, it was found that former smokers were less affected by OSA^9^. In some patient studies, the association between a history of smoking and OSA could not be substantiated at all^10–13^. A retrospective study with 3,791 patients of a sleep clinic in Thessaloniki, Greece, and another study with 733 patients of a sleep clinic in Changhua, Taiwan, found an elevated AHI in smokers, but the association became non-significant after adjustment for age, body-mass-index (BMI) and other confounders^11,12^. Population-based studies on this topic remain scarce. Using data from the Korea National Health and Examination Survey, an association between the STOP-Bang questionnaire (a screening instrument for OSA^14^ and smoking was observed^15^. Because of the absence of PSG this study lacks exact AHI values, which limits its interpretability.

The present study aimed to address the lack of population-based evidence regarding the relationship between smoking and OSA. We used data on smoking behavior, polysomnography (PSG), and relevant covariates from the TREND-0 cohort of the Study of Health in Pomerania (SHIP), a population-based survey conducted in northeastern Germany^16^. The primary objective was to examine whether current or former smoking is associated with OSA severity, as measured by AHI, while adjusting for key confounders age and BMI.

## Methods

### Sample

The study sample was a subsample of SHIP, a population-based cohort study aimed at monitoring health status in the West Pomerania region of Germany^16^. All participants gave informed consent in writing. The study was approved by the local ethics committee. As part of the initial assessment of the SHIP-TREND cohort (2008–2012), participants were invited to undergo overnight PSG in a sleep laboratory^17^. Out of the 4,420 individuals in the SHIP-TREND-0 cohort, 1,264 agreed to participate. Complete data on Apnea-Hypopnea Index (AHI) was obtained for 1,209 individuals. Of these 1,206 completed the smoking interview. No further exclusion criteria were applied - all 1,206 individuals with complete data on AHI and on smoking status were included in the analysis.

### Polysomnography

An attended, laboratory-based overnight PSG was conducted in accordance with the American Academy of Sleep Medicine (AASM)^18^ guidelines, using ALICE 5 PSG devices (Philips Respironics, Eindhoven). Recordings included six electroencephalogram (EEG) channels, two electrooculogram (EOG) channels, three electromyogram (EMG) channels (at the chin and tibialis muscles of each leg), one electrocardiogram (ECG), respiratory inductive plethysmography, a nasal pressure sensor, pulse oximetry, a snoring microphone, and a body position sensor. Sleep stages, respiratory events, and arousals were scored according to the same AASM guidelines^18^.

### Smoking

Smoking behavior was assessed through a structured interview with a specific focus on cigarette consumption^19^. Among other things, participants were asked if they ever smoked a cigarette and if applicable whether they are currently smoking, how many cigarettes they smoke per day, whether they ever smoked on a regular basis, or at what age they started or quit smoking. Smoking hiatus was not recorded - only a start date and potentially an end date were collected.

### Study variables

#### Variables of interest

Two variables of interest were introduced in separate models. For the first variable of interest “smoker”, participants were divided in current regular smokers (*N* = 208) and non-smokers as well as occasional smokers (*N* = 998, including former smokers, see below). Current regular smokers smoked at least one cigarette per day. For the second variable of interest “former smoker” current non-smoking participants were divided in former smokers (*N* = 190, excluding short term quitters, see below) and never-smokers (*N* = 495). Former smokers were defined as regular smokers for at least ten years that had quit smoking one year or longer ago^20^. Current smokers were excluded from all analyses concerning the variable “former smoker”.

#### Confounders

*Age.* In Germany, smoking behavior varies across different age groups^21^. As participants age they have more opportunity to start and eventually quit smoking and there is a minimum age where participants could have stopped smoking for 10 years or longer. Age is also an established factor in sleep apnea and is associated with musculoskeletal changes in the pharynx^22^. The regular smokers had a mean age of m = 45 years (SD = 13, range: 20–78) while the non-smokers and occasional smokers had a mean age of m = 54 years (SD = 14, range: 22–81). The former smokers had a mean age of m = 59 years (SD = 11, range: 25–80) while the never-smokers had a mean age of m = 53 years (SD = 14, range: 21–81).

*BMI.* Smoking as well as AHI is associated with BMI^23,24^. The regular smokers had a mean BMI of m = 27.5 (SD = 4.7, range: 18.5–41.8) while the non-smokers and occasional smokers had a mean BMI of m = 28.7 (SD = 5.0, range: 18.4–52.8). The former smokers had a mean BMI of m = 30.4 (SD = 4.6, range: 20.3–52.1) while the never-smokers had a mean BMI of m = 28.4 (SD = 5.0, range: 18.4–50.1).

#### Outcome

*AHI.* The regular smokers had a mean AHI of m = 10.3 (SD = 15.1, range: 0–85.9) while the non-smokers and occasional smokers had a mean AHI of m = 10.7 (SD = 14.2, range: 0–91.8). The former smokers had a mean AHI of m = 15.1 (SD = 15.9, range: 0–72.7) while the never-smokers had a mean AHI of m = 9.4 (SD = 12.9, range: 0–89.1).

Table 1 provides an overview of the descriptive statistics for the study sample.

**Table 1:** Population characteristics with total number (and percent) for the categorical variables and arithmetic mean (and standard deviation) for the continuous variables.

	Regular smoker *N* = 208	Non-smoker *N* = 998	Former smoker *N* = 190	Never smoker *N* = 495
AHI	m = 10.3 (SD = 15.1)	m = 10.7 (SD = 14.2)	m = 15.1 (SD = 15.9)	m = 9.4 (SD = 12.9)
AHI < 5: normal	*N* = 112 (54%)	*N* = 492 (49%)	*N* = 59 (31%)	*N* = 264 (53%)
5 ≤ AHI < 15: mild	*N* = 53 (25%)	*N* = 262 (26%)	*N* = 61 (32%)	*N* = 125 (25%)
15 ≤ AHI < 30: moderate	*N* = 20 (10%)	*N* = 162 (16%)	*N* = 45 (24%)	*N* = 73 (15%)
AHI ≥ 30: severe	*N* = 23 (11%)	*N* = 82 (8%)	*N* = 25 (13%)	*N* = 33 (7%)
Male	*N* = 115 (55%)	*N* = 492 (49%)	*N* = 148 (78%)	*N* = 197 (40%)
Female	*N* = 93 (45%)	*N* = 506 (51%)	*N* = 42 (22%)	*N* = 298 (60%)
Age	m = 45 years (SD = 13)	m = 54 years (SD = 14)	m = 59 years (SD = 11)	m = 53 years (SD = 14)
BMI	m = 27.5 (SD = 4.7)	BMI of m = 28.7 (SD = 5.0)	m = 30.4 (SD = 4.6)	m = 28.4 (SD = 5.0)
Obese	*N* = 67 (32%)	*N* = 351 (35%)	*N* = 99 (52%)	*N* = 161 (33%)
Non-obese	*N* = 141 (68%)	*N* = 647 (65%)	*N* = 91 (48%)	*N* = 334 (67%)

Former smoker requires regular smokers for at least ten years; former smoker and never smoker subgroup of non-smoker.

### Data analysis

For data analysis and visualization R Statistics^25^ with the packages haven^26^, dplyr^27^, MASS^28^, ggplot2^29^, brant^30^, car^31^, splines^32^, tableone^33^, and gvlma^34^ were utilized. Separate planned linear regressions were computed for an immediate and a long-term model with the confounders age and BMI. In case model assumption were violated, additional tests were conducted to validate the robustness of the results.

## Results

### Visualization

**Fig. 1:**
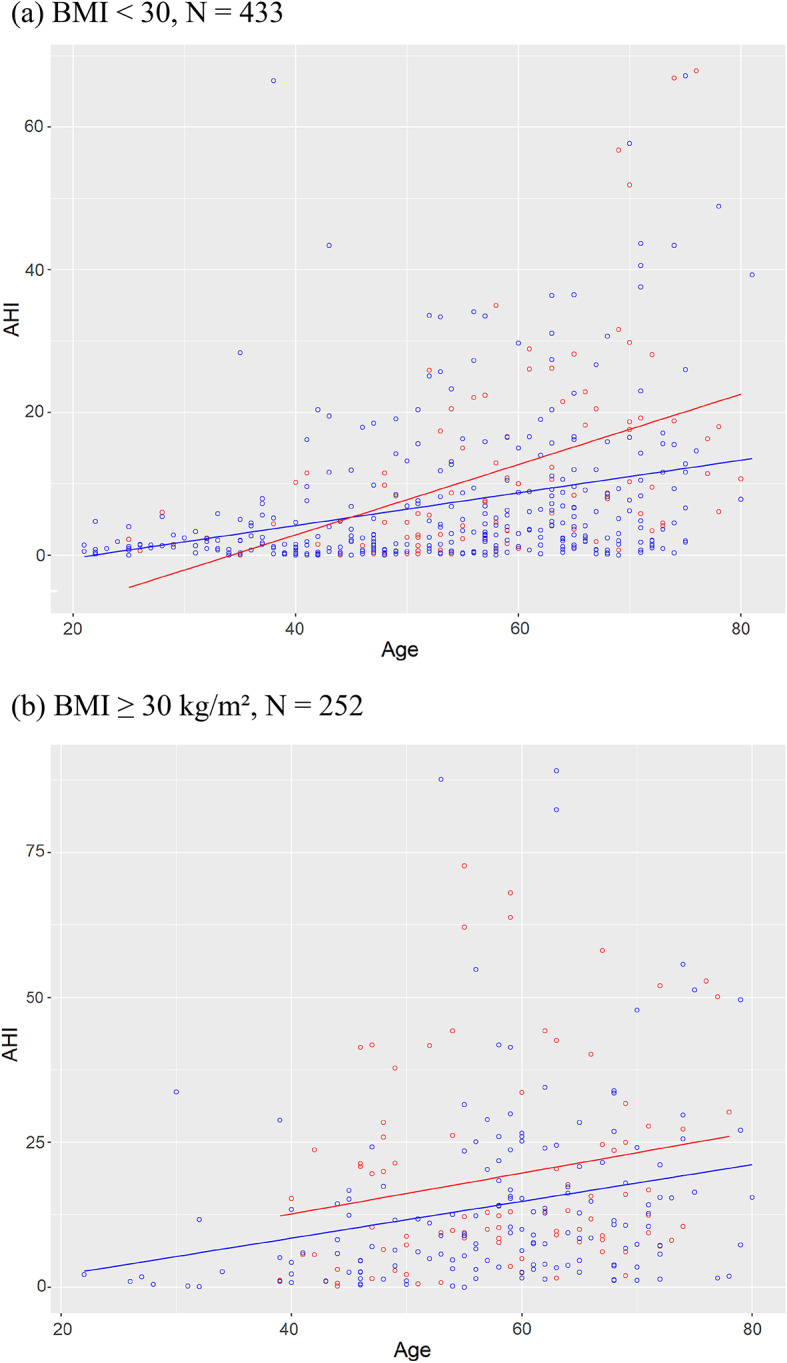
AHI according to age for smokers (red) and non-smokers (blue) for the non-obese participants (**a**) and the obese participants (**b**).

**Fig. 2:**
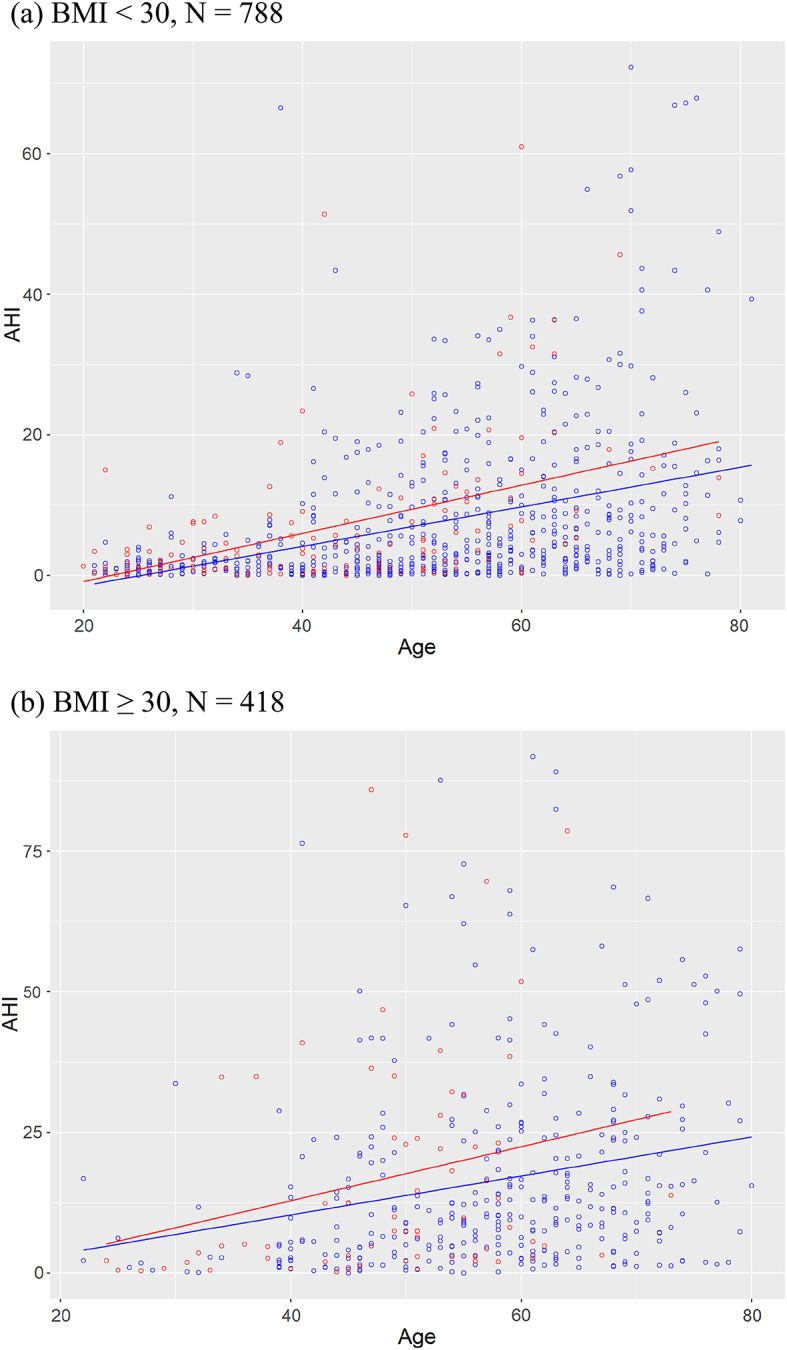
AHI according to age for the former smokers (red) and the never-smokers (blue) for the non-obese participants (**a**) and the obese participants (**b**).

Linear associations between AHI and age for the non-obese and the obese participants (BMI ≥ 30 kg/m²) were visualized for the smokers and non-smokers (Fig. 1) as well as the former smokers and the never-smokers (Fig. 2) separately.

### Regression analyses

*Smoker vs. non-smokers.* To test the association between the dichotomous variable of interest Smoker (smoker / non-smoker) and AHI, a linear regression model with the continuous confounders Age (in years) and BMI (in kg/m²) was computed. In this linear regression model the variable of interest Smoker was significantly associated with the outcome AHI, β = 3.11 [CI 1.13; 5.10], *p* = .002, however, a non-linear distribution of residuals indicated that assumptions of the model might be violated. Therefore, an ordinal logistic regression model with the same variable of interest and confounders on the AHI severity (AHI < 5: normal, 5 ≤ AHI < 15: mild, 15 ≤ AHI < 30: moderate, AHI ≥ 30: severe) was computed. Smoking status remained significantly associated with higher AHI severity categories, OR 1.75 [CI 1.27; 2.41], *p* < .001. However, a Brand-Wald-test indicated that the assumption of proportional odds might be invalidated for the model. A closer inspection of Fig. 1 reveals that smokers and non-smokers might have different centroids concerning the variable Age. Therefore, participants were split into two age groups roughly at the median (younger: age in years < 54, older: age in years ≥ 54, see Table 2) and the ordinal logistic regression was repeated for both age groups separately, yielding significant results for the variable of interest Smoker for the younger participants, OR 1.56 [CI 1.03; 2.35], *p* = .036, and the older ones, OR 1.75 [CI 1.27; 2.41], *p* < .001, as before. Keeping all other factors stable, being a regular smoker increases the chance of having a higher severity grade of sleep apnea by 56% for the younger participants and respectively by 75% for the older ones.

**Table 2:** Population characteristics of the younger and older regular smokers and non-smokers with total number (and percent) for the categorical variables and arithmetic mean (and standard deviation) for the continuous variables.

	Younger Participants (age in years < 54) *N* = 592		Older Participants (age in years ≥ 54) *N* = 614	
	Smoker *N* = 151	Non-smoker *N* = 441	Smoker *N* = 57	Non-smoker *N* = 557
AHI	m = 7.7 (SD = 13.2)	m = 6.2 (SD = 10.4)	m = 17.3 (SD = 17.6)	m = 14.3 (SD = 15.7)
AHI < 5: normal	*N* = 97 (64%)	*N* = 302 (69%)	*N* = 15 (26%)	*N* = 190 (34%)
5 ≤ AHI < 15: mild	*N* = 33 (22%)	*N* = 84 (19%)	*N* = 20 (35%)	*N* = 178 (32%)
15 ≤ AHI < 30: moderate	*N* = 11 (7%)	*N* = 41 (9%)	*N* = 9 (16%)	*N* = 121 (22%)
AHI ≥ 30: severe	*N* = 10 (7%)	*N* = 14 (3%)	*N* = 13 (23%)	*N* = 68 (12%)
Male	*N* = 94 (62%)	*N* = 237 (54%)	*N* = 39 (68%)	*N* = 279 (50%)
Female	*N* = 57 (38%)	*N* = 204 (46%)	*N* = 18 (32%)	*N* = 278 (50%)
Age	m = 39 years (SD = 10)	m = 42 years (SD = 9)	m = 60 years (SD = 6)	m = 64 years (SD = 7)
BMI	m = 27.1 (SD = 4.7)	BMI of m = 27.3 (SD = 4.7)	m = 28.7 (SD = 4.7)	m = 29.7 (SD = 4.9)
Obese	*N* = 44 (29%)	*N* = 114 (26%)	*N* = 23 (40%)	*N* = 237 (43%)
Non-obese	*N* = 107 (71%)	*N* = 327 (74%)	*N* = 34 (60%)	*N* = 320 (57%)

Following a reviewer suggestion, a linear regression model including age, BMI, sex, and pack-years as confounders, with restricted cubic splines (4 knots) for age and BMI was fitted, yielding a consistent association, β = 2.59 [CI 0.41; 4.77], *p* = .02.

*Former smoker vs. never-smoker.* Similarly to analysis of *smoker vs. non-smoker*, a linear regression model with the continuous confounders Age (in years) and BMI (in kg/m²) on AHI (apnea and hypopnea events/h) for the dichotomous variable of interest Former Smoker (former smoker / never-smoker) was computed. The variable of interest Former Smoker was significantly associated with the outcome AHI, β = 3.13 [CI 0.99; 5.28], *p* = .004, but a non-linear distribution of residuals indicated that assumptions of the model might not be fulfilled. Therefore, an ordinal logistic regression model on the AHI severity (AHI < 5: normal, 5 ≤ AHI < 15: mild, 15 ≤ AHI < 30: moderate, AHI ≥ 30: severe) for the same predictor and confounders as before was computed, resulting in a significant association between Former Smoker and AHI severity, OR 1.76 [CI 1.27; 2.43], *p* < .001, indicating that compared to the never-smoker a former smoker had a 76% higher chance of reaching a higher severity grade of sleep apnea, given both other factors were held stable. To allow for an analogue interpretation groups were split as for the smokers vs. non-smokers at the median age (younger: age in years < 57, older: age in years ≥ 57, see Table 3). For the younger participants, the association between AHI and former smoker status failed the conventional level of significance OR 1.49 [CI 0.89; 2.49], *p* = .13, whereas in the older participants the association was significant, OR 1.91 [CI 1.26; 2.91], *p* = .002, resulting in a higher relative risk of 49% or of 91% being in the group with the higher severity grade.

**Table 3:** Population characteristics of the younger and older former smokers and never smokers with total number (and percent) for the categorical variables and arithmetic mean (and standard deviation) for the continuous variables.

	Younger Participants (age in years < 57) *N* = 328		Older Participants (age in years ≥ 57) *N* = 357	
	Former smoker *N* = 82	Never smoker *N* = 246	Former smoker *N* = 108	Never smoker *N* = 249
AHI	m = 11.0 (SD = 14.4)	m = 6.3 (SD = 10.6)	m = 18.3 (SD = 16.3)	m = 12.5 (SD = 14.1)
AHI < 5: normal	*N* = 40 (49%)	*N* = 165 (67%)	*N* = 19 (18%)	*N* = 99 (40%)
5 ≤ AHI < 15: mild	*N* = 20 (24%)	*N* = 51 (21%)	*N* = 41 (38%)	*N* = 74 (30%)
15 ≤ AHI < 30: moderate	*N* = 15 (18%)	*N* = 21 (9%)	*N* = 30 (28%)	*N* = 52 (21%)
AHI ≥ 30: severe	*N* = 7 (9%)	*N* = 9 (4%)	*N* = 18 (17%)	*N* = 24 (10%)
Male	*N* = 57 (70%)	*N* = 119 (48%)	*N* = 91 (84%)	*N* = 78 (31%)
Female	*N* = 25 (30%)	*N* = 127 (52%)	*N* = 17 (16%)	*N* = 171 (69%)
Age	m = 47 years (SD = 7)	m = 43 years (SD = 9)	m = 66 years (SD = 6)	m = 66 years (SD = 6)
BMI	m = 30.0 (SD = 4.9)	BMI of m = 27.5 (SD = 5.0)	m = 30.8 (SD = 4.4)	m = 29.2 (SD = 4.9)
Obese	*N* = 39 (48%)	*N* = 65 (26%)	*N* = 52 (48%)	*N* = 96 (39%)
Non-obese	*N* = 43 (52%)	*N* = 181 (74%)	*N* = 56 (52%)	*N* = 153 (61%)

Former smoker requires regular smokers for at least ten years; former smoker and never smoker subgroup of non-smoker.

As before, following a reviewer suggestion, a linear regression model including age, BMI, sex, and pack-years as confounders, with restricted cubic splines (3 knots) for age and BMI was fitted, yielding β = 2.50 [CI − 0.67; 5.67], *p* = .12.

## Discussion

Concerning current smokers, our findings align with the current meta-analysis by Zeng et al.^8^, as we found a substantial association with OSA with an increased relative risk of a higher severity grade of 56% for the younger and 75% for the older participants. Likewise, our findings are in line with the meta-analysis regarding former smokers with a nearly identical relative risk of 76%. Looking at the relative risk for age groups separately, a relative higher risk of 49% is found in the younger and of 91% in the older participants.

These findings are in line with several pathophysiological mechanisms explaining the association between smoking and increased OSA severity. Cigarette smoke promotes upper airway inflammation and oxidative stress, narrowing the airway and increasing its collapsibility during sleep^35,36^. Structural changes elevate airway resistance and exacerbate upper airway obstruction in smokers with OSA^37^. Moreover, smoking amplifies OSA related hypoxia, promoting systemic inflammation and further airway dysfunction^38^.

### Strength and limitations

This study is based on a large population-based cohort with objective assessment of OSA using full-night polysomnography, reducing selection bias and improving generalizability in contrast to patient samples. Participation in polysomnography was voluntary, and individuals with sleep-related symptoms such as snoring may be overrepresented; however, there is no indication that this resulted in systematic bias in AHI distribution^5,39^. Detailed smoking data allowed differentiation between current, former, and never smokers. Adjustment for key confounders and using alternative modeling approaches support the robustness of the findings. Analyses of former versus never smokers may have been underpowered duo to limited number of the later. Residual and unmeasured confounding cannot be excluded. The cross-sectional design precludes causal inference.

### Conclusion

Although the detrimental effects of smoking are well established, the present findings might paradoxically be interpreted as discouraging cessation, potentially fostering a fatalistic attitude that the damage is already done once smoking becomes habitual. However, it is important to note here that our results concern long term smokers after an indeterminable time (but at least a year) of non-smoking. Shorter smoking histories and longer periods of cessation might lead to more beneficial outcomes. Furthermore, our current study and most studies included in aforementioned meta-analysis were retrospective or cross-sectional in nature, limiting a causal interpretation that smoking contributes to the development or progression of OSA. Given the lower odds ratios observed in the younger participants, one might cautiously speculate that this age group exhibits greater resilience concerning OSA in smokers or hope for more regenerative capacity in response to the long-term effects of smoking on OSA. Taking into account that continued smoking is detrimental to other health factors, too, of which at least some are partially reversible, there seems to be no reason to not recommend smoking cessation^1,20,40^. Yet, there is a problem. Smoking cessation might lead to weight gain, which in turn increases cardiovascular risk^41^. But for OSA, weight-loss management is recommended to reduce the AHI and the cardiovascular risks associated with obesity (and OSA for that matter)^42,43^. While smoking cessation and weight management should both be considered key components of care in smokers with OSA, addressing both simultaneously may be overwhelming for patients. Prioritization should therefore be guided by individual patient circumstances and preferences^44^. The only way to win this game is to not start smoking in the first place.

## Data Availability

The data that support the findings of this study are available from the Transferstelle für Daten- und Biomaterialienmanagement [Office for transfer of data and bio materials] of the University Medicine Greifswald, Study of Health in Pomerania (SHIP: https://transfer.ship-med.uni-greifswald.de/FAIRequest/). Access is restricted and needs approval of the board.
